# Differential Ligand Binding Affinities of Human Estrogen Receptor-α Isoforms

**DOI:** 10.1371/journal.pone.0063199

**Published:** 2013-04-30

**Authors:** Amanda H. Y. Lin, Rachel W. S. Li, Eva Y. W. Ho, George P. H. Leung, Susan W. S. Leung, Paul M. Vanhoutte, Ricky Y. K. Man

**Affiliations:** Department of Pharmacology and Pharmacy, Li Ka Shing Faculty of Medicine, The University of Hong Kong, Pokfulam, Hong Kong SAR; Florida International University, United States of America

## Abstract

Rapid non-genomic effects of 17β-estradiol are elicited by the activation of different estrogen receptor-α isoforms. Presence of surface binding sites for estrogen have been identified in cells transfected with full-length estrogen receptor-α66 (ER66) and the truncated isoforms, estrogen receptor-α46 (ER46) and estrogen receptor-α36 (ER36). However, the binding affinities of the membrane estrogen receptors (mERs) remain unknown due to the difficulty of developing of stable mER-transfected cell lines with sufficient mER density, which has largely hampered biochemical binding studies. The present study utilized cell-free expression systems to determine the binding affinities of 17β-estradiol to mERs, and the relationship among palmitoylation, membrane insertion and binding affinities. Saturation binding assays of human mERs revealed that [^3^H]-17β-estradiol bound ER66 and ER46 with K_d_ values of 68.81 and 60.72 pM, respectively, whereas ER36 displayed no specific binding within the tested concentration range. Inhibition of palmitoylation or removal of the nanolipoprotein particles, used as membrane substitute, reduced the binding affinities of ER66 and ER46 to 17β-estradiol. Moreover, ER66 and ER46 bound differentially with some estrogen receptor agonists and antagonists, and phytoestrogens. In particular, the classical estrogen receptor antagonist, ICI 182,780, had a higher affinity for ER66 than ER46. In summary, the present study defines the binding affinities for human estrogen receptor-α isoforms, and demonstrates that ER66 and ER46 show characteristics of mERs. The present data also indicates that palmitoylation and membrane insertion of mERs are important for proper receptor conformation allowing 17β-estradiol binding. The differential binding of ER66 and ER46 with certain compounds substantiates the prospect of developing mER-selective drugs.

## Introduction

Rapid non-genomic actions of estrogen are physiologically significant in our biological systems including the cardiovascular, nervous and skeletal systems [1], [2]. Short incubation of 17β-estradiol (the major active form of estrogen) rapidly triggers the formation of intracellular signaling molecules such as cAMP [3], [4], cGMP [5] and calcium [6], leading to rapid cellular responses by activation of subsequent signaling pathways, such as protein kinase A, protein kinase C and extracellular regulated kinase (ERK) [2], [7]. For example, physiological concentrations of 17β-estradiol enhanced endothelium-dependent relaxations induced by acetylcholine in the rat aorta [8]. This response is mediated by activation of the phosphatidylinositol 3-kinase (PI3K)/Akt pathway and endothelial nitric oxide synthase (eNOS) and is regulated by a non-receptor tyrosine kinase c-Src [9]–[11]. This type of rapid (within seconds to a few minutes) response to estrogen is non-genomic, since it does not involve gene transcription and protein synthesis [12].

The estrogen receptors (ER), ERα and ERβ, are well recognized as nuclear steroid receptors that interact with specific DNA sequences, namely estrogen responsive elements (ERE), to regulate gene expression in response to estrogen [13]. The existence of membrane estrogen receptors (mERs), responsible for the non-genomic actions of estrogen, was first indicated by the presence of specific surface binding sites for estrogen conjugated with cell-impermeable albumin [14]. Immunological studies using anti-ERα and ERβ antibodies have detected ERs in both nuclear and cell membrane fractions of cells endogenously expressing or transfected with ERα or ERβ [15], [16]. Endothelial cells from ERα and ERβ homozygous double knock-out mice lose the ability to mediate rapid estrogen signaling, and ERα and ERβ are not expressed in either nuclear and membrane cell fractions of these animals [17]. Membrane and nuclear cell fractions of ERα-transfected CHO cells bind estrogen with similar affinities, but the membrane receptor number of ER66 was estimated to be only about 3% of the total nuclear receptor density [16]. These data show that ERα and ERβ or their isoforms are essential in rapid estrogen signaling, and also suggest that the putative mER is a homologue of the classical nuclear estrogen receptor-α, also named estrogen receptor-α66 (ER66) in view of its molecular weight. Two truncated splice variants of the ERα, 46 kDa estrogen receptor (ER46) [18] and 36 kDa estrogen receptor (ER36) [19] have been identified as mERs. To our knowledge, molecular identities of membrane isoforms of another estrogen receptor homologue, ERβ, have not yet been reported.

Functions of mERs are dependent on palmitoylation and membrane localization. Translocation of ER66 to plasma membrane as mER is achieved by interaction with the scaffolding protein of caveolae, caveolin-1 [20]. This interaction of ER66 with caveolin-1 is palmitoylation-dependent. Point mutation of Cys447 residue of ER66 to Ala impairs ER66 palmitoylation and membrane localization, and hence the subsequent rapid estrogen signaling pathways mediated by the membrane-localized ER66 [21], [22]. The truncated splice variant, ER46, has lost the AF-1 transactivation domain, but retains domains for palmitoylation and caveolin-1 association [18], [22]. Loss of the AF-1 domain has a minimal influence on the ability of ER46 to elicit non-genomic estrogenic responses, but also enhances palmitoylation over wild-type ER66 [22], [23]. This suggests that a larger number of ER46 is palmitoylated and translocated to the membrane compared to ER66. In line with this suggestion, ER46 mediates estrogen-induced eNOS activation in a more efficient manner than ER66 [24]. Another splice variant of ER66, ER36, is devoid of the AF-1 and AF-2 transactivation domains and part of the ligand binding domain in the C-terminal is replaced by an unique 27 amino acid sequence [19], ER36 mediates the stimulation by 17β-estradiol of mitogen-activated protein kinase (MAPK) pathway [25]. ER36 also mobilizes intracellular calcium when acutely stimulated by 17β-estradiol [26].

Although the functional responses elicited by the mERs have been studied extensively [24], [25], [27], [28] and surface binding sites of 17β-estradiol on plasma membrane of ER66, ER46 or ER36 expressing cells was shown by confocal microscopy [27], [29], their binding affinities towards estrogen have not yet been determined. Biochemical binding studies were greatly hampered by the difficulty in developing stable mER-transfected cell line and the relatively low expression level of mER on the plasma membrane [16], [30], [31]. The present experiment determined the binding affinities of estrogen to human membrane isoforms of ERα, taking the advantages of cell-free expression systems. The importance of palmitoylation, translocation of mERs and membrane insertion in affecting these binding affinities was also investigated. Finally, the relative binding affinities of different mERs to various estrogen receptor agonists and antagonists, including phytoestrogens, were evaluated.

## Results

### ER66, ER46 and ER36 Colocalize with Plasma Membrane

**Figure 1:**
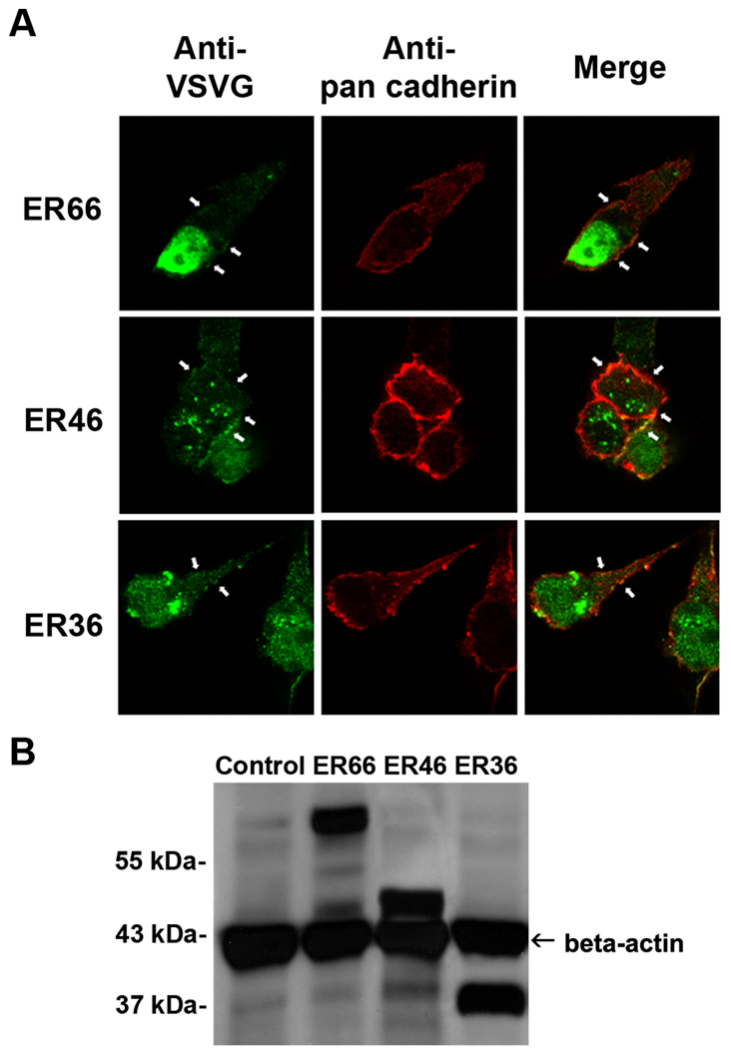
Colocalization of ER66, ER46 and ER36 with plasma membrane. (**A**) Confocal microscopy of HEK293 cells transfected with VSVG-tagged ER66, ER46 or ER36 treated with anti-VSVG followed by Oregon Green 488 goat anti-rabbit secondary antibody (green). Plasma membrane is marked with anti-pan cadherin primary antibody followed by Texas Red goat anti-mouse antibody (red). Overlay images show colocalization of ER66, ER46 and ER36 with the plasma membrane (yellow). White arrows indicate some colocalization sites. (**B**) Western blot showing relative transfection efficiencies of His-tagged ER66, ER46 and ER36 in HEK293 cells using β-actin as a reference. Bands of 66, 46 and 36 kDa for ERs were detected by anti-HisG antibody and bands of 42 kDa were detected by anti-β-actin antibody.

Vesicular stomatitis virus glycoprotein (VSVG)-tagged ER66, ER46 and ER36 proteins were transfected to HEK293 cells with similar transfection efficiencies (Fig. 1). Anti-pan cadherin was used as plasma membrane marker. Confocal microscopy revealed that ER66 were expressed dominantly in the nuclear region but that a small percentage was expressed on the plasma membrane as shown by the colocalization with the plasma membrane marker, pan cadherin. ER46 and ER36 were expressed mainly in the cytosol and only a small portion on the plasma membrane.

### Expression of mER Proteins Using Cell-free Expression Systems

**Figure 2:**
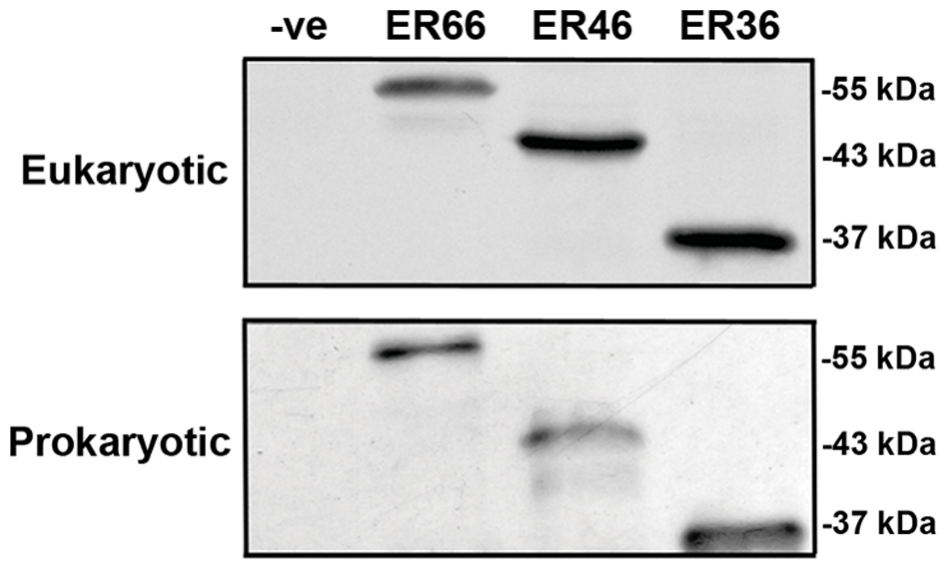
Expression of ER66, ER46 and ER36 in cell free expression systems. Expression of ER66, ER46 and ER36 in eukaryotic and prokaryotic expression systems composed of cell lysates from rabbit reticulocytes or *E.coli,* respectively. Bands of 66, 46 and 36 kDa, corresponding for ER66, ER46 and ER36, respectively, were detected by anti-HisG antibody using Western blotting.

Human ER66, ER46 and ER36 proteins were expressed in both eukaryotic and prokaryotic expression systems. The eukaryotic and prokaryotic expression systems are *in vitro* protein expression systems composed of cell lysates from rabbit reticulocyte and *E. coli*, respectively [32]. The rabbit reticulocyte lysate contains a large amount of heat-shock proteins, which function as molecular chaperones to ensure proper receptor folding [33]. Immunoreactive bands of molecular sizes of 66, 46 and 36 kDa were detected by anti-HisG antibody (Fig. 2), which corresponds to the molecular sizes of the His-tagged receptor proteins.

### Serum Levels of 17β-estradiol Bind to ER66 and ER46, but not to ER36

**Figure 3:**
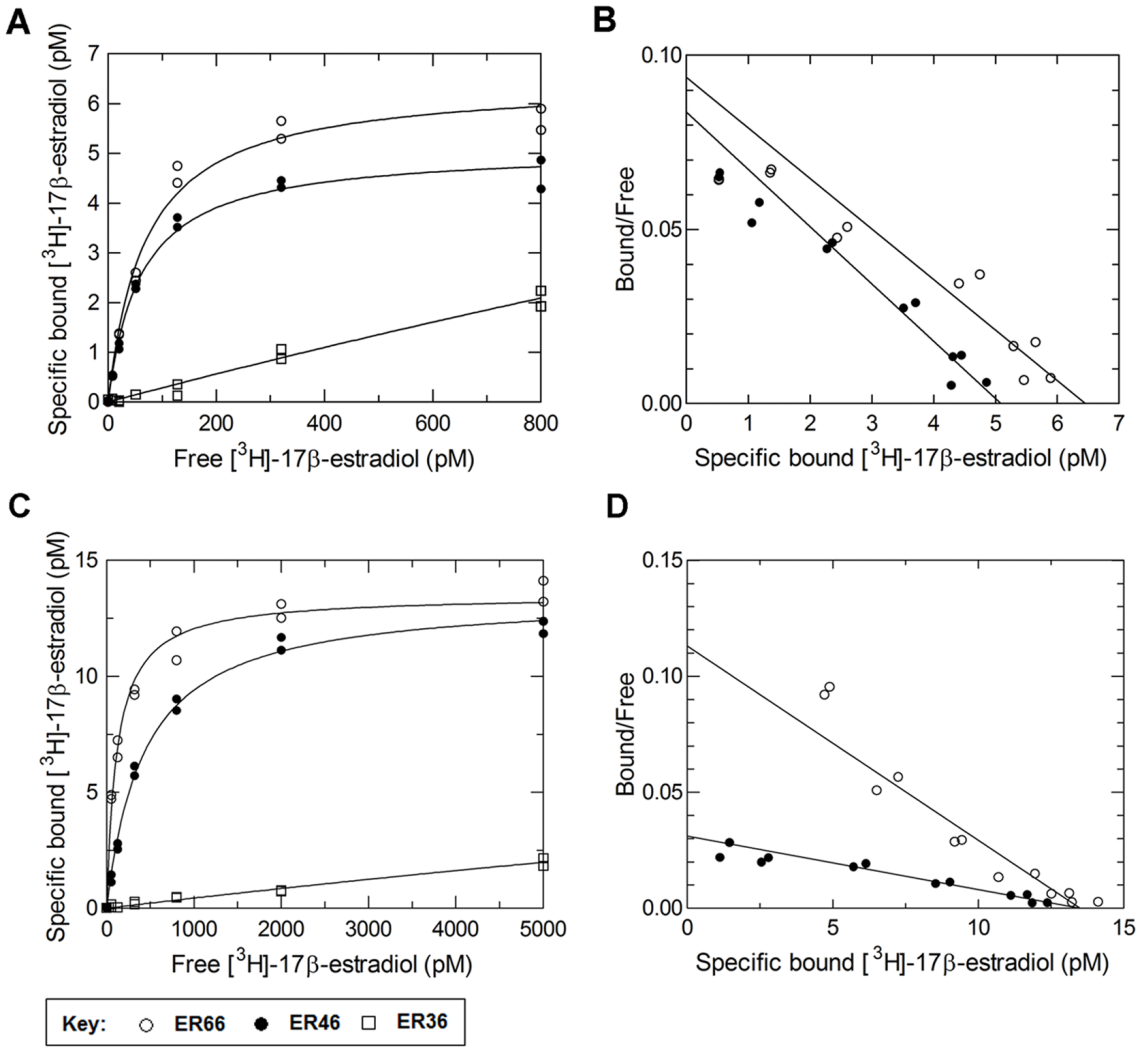
ER66 and ER46, but not ER36, binds with 17β-estradiol at physiological concentrations. (**A**) Specific binding of [^3^H]-17β-estradiol to ER66 (○) and ER46 (•) expressed in eukaryotic system with K_d_ values of 68.8 pM and 60.72 pM, respectively. No saturable specific binding was found for ER36 (□). (**C**) Specific binding of [^3^H]-17β-estradiol to ER66 (○) and ER46 (•) expressed in prokaryotic system with K_d_ values of 119.4 pM and 433.7 pM, respectively. No saturable specific binding was found for ER36 (□). (**B, D**) Scatchard plot analysis for ER66 and ER46 showed single-site binding. Results are representative of three experiments each done in duplicate.

ER66, ER46 and ER36 were expressed in the eukaryotic cell-free expression system. Saturation binding assays demonstrated that [^3^H]-17β-estradiol bound to ER66 and ER46 specifically with an equilibrium dissociation constant (K_d_) of 68.8 pM and 60.7 pM, respectively, whereas ER36 showed no saturable specific binding (Fig. 3A). Scatchard plots revealed a single population of binding sites for [^3^H]-17β-estradiol in ER66 and ER46 (Fig. 3B).

Saturation binding assays showed that the K_d_ values of 17β-estradiol binding for ER66 and ER46 expressed in prokaryotic system were 119.4 pM and 433.7 pM, respectively, whereas ER36 showed no saturable specific binding (Fig. 3C). Scatchard plots showed that 17β-estradiol bound to ER66 and ER46 at a single binding site (Fig. 3D). The binding affinities of 17β-estradiol to ER isoforms are summarized in Table 1.

**Table 1:** Summary Table of binding affinities of 17β-estradiol to mERs.

	K_d_ of ER66	K_d_ of ER46	K_d_ of ER36
Eukaryotic expression system (with NLP)	68.8 pM	60.7 pM	no specific binding
Prokaryotic expression system (with NLP)	119.4 pM	433.7 pM	no specific binding
Eukaryotic expression system (with NLP and 2-bromopalmitate)	185.0 pM	337.5 pM	–
Eukaryotic expression system (without NLP)	130.4 pM	399.7 pM	–

### Inhibition of Posttranslational Palmitoylation and Membrane Insertion of mERs Impaired Ligand Binding

**Figure 4:**
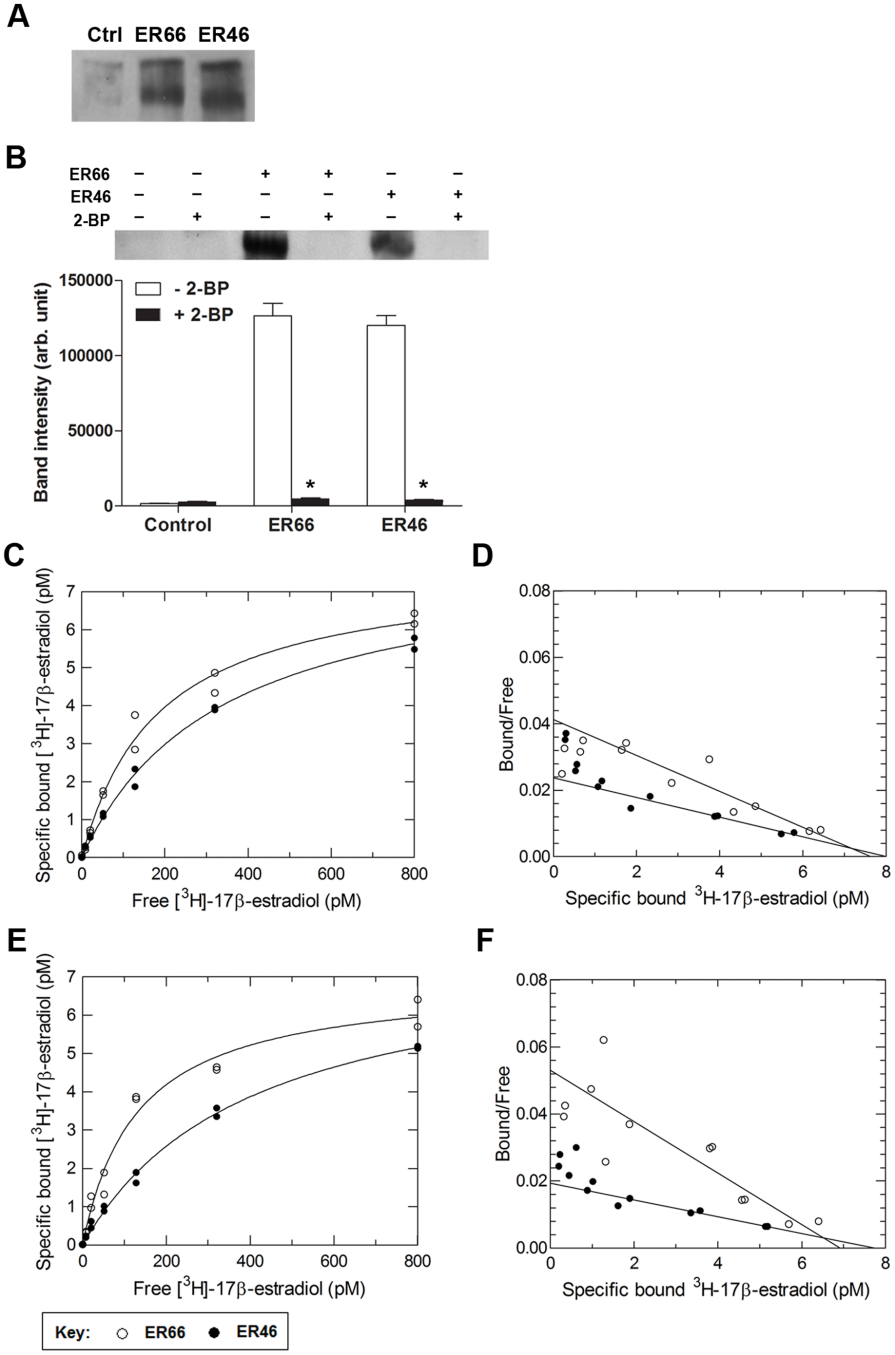
Palmitoylation and membrane insertion is important for mER binding. (**A**) ER66 and ER46 proteins were analyzed by native gel electrophoresis. Non-palmitoylated and palmitoylated ER proteins were separated as upper and lower immunoreactive bands to anti-HisG antibody due to their difference in net electric charge imposed by the palmitic acid group in palmitoylated proteins. (**B**) Representative blot (upper panel) and quantitative band intensity data (lower panel) showing the effect of 2-bromopalmitate (2-BP) treatment on the expression of palmitoylated ERs. Data shown are the mean ± S.E. (n = 8–9) and *p<0.05. Specific binding of [^3^H]-17β-estradiol to ER66 (○) and ER46 (•) expressed in eukaryotic system (**C**) in the presence of 100 µM 2-BP or (**E**) in the absence of the membrane substitute, nanolipoprotein particles (NLPs). When expressed in the presence of the palmitoylation inhibitor, the binding affinities of [^3^H]-17β-estradiol to ER66 and ER46 were reduced to 185 pM and 337.5 pM, respectively. In the absence of NLPs, the binding affinities of [^3^H]-17β-estradiol to ER66 and ER46 were reduced to 130.4 pM and 399.7 pM, respectively. (**D, F**) Scatchard plot analysis revealed a single population of binding sites for 17β-estradiol to both ER66 and ER46. Results are representative of three experiments each done in duplicate.

In order to elucidate whether or not palmitoylation is involved in ligand binding of mERs, a palmitoylation inhibitor (2-bromopalmitate) was added to the transcription/translation reaction in the eukaryotic cell-free expression system. Non-palmitoylated and palmitoylated ER66 or ER46 were separated by native protein electrophoresis at neutral pH, which distinguishes proteins according to their size, shape and intrinsic charge. Upper and lower bands were detected in cell lysates expressing ER66 or ER46 (Fig. 4A). Addition of the palmitic acid group decreases the positive electric charge of ER66 and ER46. Therefore, palmitoylated ER proteins are represented by the lower bands, as proteins more negatively charged migrate faster towards the positive electrode. Treatment with 2-bromopalmitate (100 µM) abolished the expression of palmitoylated ER66 and ER46 (Fig. 4B). Inhibition of palmitoylation by 2-bromopalmitate reduced the binding affinities of ER66 and ER46 to K_d_ values of 185 pM and 337.5 pM, respectively (Fig. 4C). Scatchard plots showed single-site binding of 17β-estradiol to ER66 and ER46 in the presence of 2-bromopalmitate (Fig. 4D).

Absence of the membrane substitute, nanolipoprotein particles (NLPs), reduced the K_d_ values of ER66 and ER46 to 130.4 pM and 399.7 pM, respectively (Fig. 4E). Scatchard plots showed single-site binding of 17β-estradiol to ER66 and ER46 in the absence of NLPs (Fig. 4F).

### Differential Binding of ER66 and ER46 to Various Estrogen Receptor Agonists and Antagonists, and Phytoestrogens

**Figure 5:**
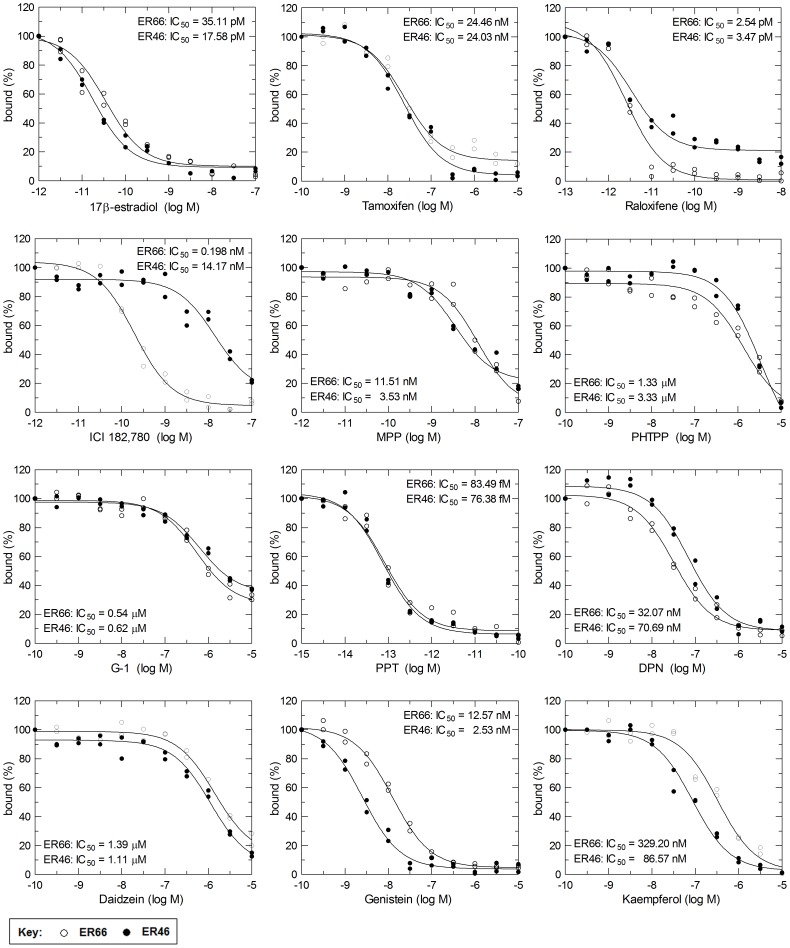
Competitive [^3^H]-17β-estradiol binding study of estrogenic compounds and phytoestrogens to ER66 or ER46. ER66 (○) or ER46 (•) protein was incubated with serial dilutions of non-radioactive estrogenic compounds or phytoestrogens at 4°C overnight in the presence of 100 pM [^3^H]-17β-estradiol. Bound and free radioligands were separated by filtration and IC_50_ values were estimated by fitting the data to a nonlinear four-parameter logistic model. Results are representative of three experiments each done in duplicate.

Equilibrium binding of [^3^H]-17β-estradiol in the presence of various estrogen receptor agonists and antagonists, and phytoestrogens, was studied to determine the relative binding affinities (RBA) for ER66 and ER46. Monophasic curves were obtained for all the compounds tested (Fig. 5). RBA values were calculated based on the IC_50_ (Table 2). The overall order of affinity of the test compounds to ER66 was: PPT>raloxifene >17β-estradiol>ICI 182,780> MPP> genistein>tamoxifen>DPN>kaempferol>G-1>PHTPP = daidzein. The order of affinity of the test compounds to ER46 was: PPT>raloxifene >17β-estradiol>genistein>MPP> ICI 182,780> tamoxifen>DPN>kaempferol>G-1> daidzein >PHTPP. ICI 182,780 had a significantly lower affinity to ER46 than ER66. The binding affinities of estrogen receptor agonists (PPT, DPN and G-1) to ER66 were similar to those to ER46. The binding affinities of selective estrogen receptor antagonists (tamoxifen and raloxifene) to ER46 were less (by half) than those to ER66. The phytoestrogens genistein and kaempferol bound to ER46 with higher affinities when compared to ER66, but the affinities of daidzein to ER66 and ER46 were approximately the same.

**Table 2:** Relative binding affinity of various estrogenic compounds and phytoestrogens to ER66 and ER46.

Compound	RBA*	
	ER66	ER46
17β-estradiol	100	100
Tamoxifen	0.144	0.073
Raloxifene	1382	507
ICI 182,780	17.73	0.124
MPP	0.305	0.498
PHTPP	0.003	0.001
G-1	0.007	0.003
PPT	42052	23016
DPN	0.109	0.025
Daidzein	0.003	0.002
Genistein	0.279	0.695
Kaempferol	0.011	0.020

* Relative binding affinity (RBA) was calculated as ratio of IC_50_ values of 17β-estradiol and compounds tested. The RBA value for estrogen was arbitrarily set at 100.

## Discussion

The major findings of the present study are: (a) ER66, ER46 and ER36 colocalize with the plasma membrane; (b) 17β-estradiol binds to ER66 and ER46 with a K_d_ value of 68.8 pM and 60.7 pM, but did not specifically bind to ER36; (c) Posttranslational palmitoylation and membrane insertion affect the binding affinities of estrogen to ER66 and ER46; and (d) ER66 and ER46 displayed differential binding affinities with various estrogen receptor agonists and antagonists, and with phytoestrogens.

The present results showed that ER66, ER46 and ER36 colocalize with the plasma membrane when transfected to HEK293 cells. However, only a small proportion of ER66, ER46 and ER36 are expressed on the plasma membrane of the cells, while the receptors mainly reside in the nucleus for ER66 and the cytosol for ER46 and ER36. In preliminary studies (data not shown), stable transfection of ERs in mammalian cells results in cell toxicity [30], [31]. ER-transfected cells stop growing and lyse after exposure to low estrogen concentrations. Therefore, ER-expressing cells have relatively low levels of the receptors [34], particularly the notoriously hard-to-express membrane receptors. These difficulties in the generation of mER-expressing stable cell lines have hindered structural and biochemical studies of mERs.

In the present study, a eukaryotic cell-free expression system composed of rabbit reticulocyte lysate was used. mERs were successfully expressed in a substantial amount for receptor ligand binding assays. Rabbit reticulocyte lysate has been widely used in studies of steroid binding and contains large amount of heat-shock proteins that functions as molecular chaperones to ensure proper folding of receptors [33], [35]. Moreover, NLPs, nanometer-sized, discoidal particles comprising amphipathic helical scaffold proteins that wrap themselves around the planar circumference of a lipid bilayer, were used to mimic the structure of the plasma membrane [32], [36], [37]. This provided an appropriate lipid bilayer plane for the attachment of ER66, ER46 and ER36.

Reference serum levels of adult female range from approximately 100 to 700 pmol/L depending on the menstrual cycle [38]. The present results demonstrate that concentrations of 17β-estradiol found in serum specifically bind to ER66 and ER46, but not to ER36. The measured K_d_ value of ER66 (68.8 pM) is in agreement with previous reports of cytosolic ER66 which ranged from 10 pM to 1 nM [39], showing the high sensitivity and validity of the current assay. ER46 is an alternative splice variant of the ER66 transcript. It is devoid of the first 173 amino acids (A and B domain) of ER66 [18]. ER46 shares the same ligand binding domain as ER66, which may explain its similar binding affinity to 17β-estradiol. On the other hand, ER36 shares a common overall structure with ER46, except that the last 138 amino acids (part of E domain and F domain) are replaced by a unique 27 amino acid domain [19]. This unique amino acid sequence in ER36 must alter the ligand binding domain, which explains why ER36 has a much different binding affinity. Previous studies showed that ER36 binds with 17β-estradiol with a K_d_ of 2.2 nM [26]. This concentration is much higher than physiological serum estrogen levels, which suggests that ER36 possesses functions other than solely to act as a mER. In line with this interpretation, ER36 activates the 17β-estradiol-induced MAPK pathway in ER36-transfected cells [25]. However, the MAPK pathway in these cells can also be activated to a similar level by the same concentrations of the inactive isomer of estrogen, 17α-estradiol, and by testosterone [25], [28]. This illustrates that the MAPK activation in ER36-transfected cells is not specific to 17β-estradiol.

The prokaryotic expression system lacks the posttranslational modifications present in eukaryotes, and thus can be used prospectively to examine the role of such modifications of mERs in 17β-estradiol binding. ER66 and ER46 expressed in prokaryotic system had lower binding affinities to 17β-estradiol than those expressed in eukaryotic system (Table 1), suggesting that posttranslational modifications are essential to proper receptor conformation of ERs for estrogen binding. Mutational analysis on palmitoylation sites of ER66 and ER46 show that membrane localization of mERs depends on palmitoylation [22], [24]. 2-Bromopalmitate was used to inhibit palmitoylation of ER66 and ER46 expressed in eukaryotic expression system. The binding affinities of non-palmitoylated ER66 and ER46 were reduced to values that are similar to ER66 and ER46 expressed in prokaryotic system. Therefore, the present data suggest that palmitoylation is the major posttranslational modification to achieve a proper conformation of mERs for estrogen binding.

Non-palmitoylable mERs mutant cannot associate with the plasma membrane [22], [24]. Hence, mERs expressed in eukaryotic system in the absence of the membrane substitute, NLPs, were used to study the significance of membrane localization. Removal of membrane substitutes reduced the binding affinities of ER66 and ER46. This provides further evidence that membrane localization is critical for proper estrogen binding. Only a small portion of ER66 can be inserted in the plasma membrane in native cells [40]. Nevertheless, the present data demonstrate that posttranslational palmitoylation and removal of NLPs have a larger influence on the binding affinities of ER46 (seven fold) than that of ER66 (two fold), implying that ER46 may depend more on membrane association to obtain correct conformation for binding and that it thus may be the predominant mER. Collectively, the present results indicate that ERs undergo posttranslational palmitoylation for translocation and insertion into the plasma membrane, and that this is crucial for proper receptor conformation for estrogen binding.

Previous *in vitro* studies concerning non-genomic vascular actions of estrogen and genistein in arteries suggested differential binding affinities of ER66 and ER46 [41], [42]. Therefore, the relative binding affinities of ER66 and ER46 towards various estrogen receptor agonists and antagonists, and phytoestrogens were studied. ICI 182,780 is a steroidal estrogen receptor antagonist that competitively binds to the ER66 [43]. MPP is an antagonist which displays 200-fold selectivity for ERα over ERβ [44], while PHTPP is a selective ERβ antagonist [45]. PPT, DPN and G-1 are selective ERα, ERβ and G protein-coupled receptor 30 (GPR30) agonists, respectively [11], [46], [47]. The selective estrogen receptor modulators, such as tamoxifen and raloxifene, act on ERs and possess tissue-specific agonistic or antagonistic effects [48]. Phytoestrogens, such as daidzein, genistein and kaempferol, exert non-genomic vascular effects in a similar manner as estrogen [42], [49], [50]. In the present study, the ligand binding affinities of the tested compounds to ER66 were in general similar to those to ER46, given that ER66 and ER46 have similar binding properties. An exception is ICI 182,780, which has a 70 times lower affinity for ER46 than for ER66. The lower affinity of ICI 182,780 for ER46 than to ER66 may account for the inability of ICI 182,780 to inhibit the non-genomic vascular actions elicited by estrogen and genistein in previous studies [41], [42]. However, these non-genomic vascular effects can be inhibited by MPP [42], which has a four-fold higher affinity for ER46 when compared to ICI 182,780. Therefore, MPP or chemical compounds with similar structural features may used as a ER46-selective antagonist.

In conclusion, the present study demonstrates the binding affinities of 17β-estradiol to human ER-α isoforms. Moreover, the present results indicate that palmitoylation and membrane insertion of ER66 and ER46 are important for proper receptor conformation for 17β-estradiol binding. Furthermore, differential binding of ER66 and ER46 with various estrogen receptor agonists and antagonists, and phytoestrogens were observed. Agonists that are ER46-selective are potential substitutes for estrogen to reduce the incidence of cardiovascular diseases [51], as they can avoid feminization in men and have lower risks in estrogen-responsive cancers.

## Materials and Methods

### Chemicals

Phosphate buffered saline (PBS) was composed of 137 mM NaCl, 2.68 mM KCl, 1.47 mM KH_2_PO_4_ and 8.1 mM Na_2_HPO_4_. [^3^H]-17β-estradiol (specific activity = 110–170 Ci/mmol) was purchased from PerkinElmer (Boston, MA, USA). 17β-estradiol, 2-bromopalmitate (2-bromohexadecanoic acid), raloxifene hydrochloride, tamoxifen, daidzein, genistein and kaempferol were obtained from Sigma-Aldrich Co. (St. Louis, MO, USA). 7a,17b-[9-[(4,4,5,5,5-Pentafluoropentyl)sulfinyl]nonyl]estra-1,3,5(10)-triene-3,17-diol (ICI 182,780), methyl-piperidino-pyrazole dihydrochloride (MPP), 4-[2-phenyl-5,7-bis(trifluoro methyl)pyrazolo[1,5-a]pyrimidin-3-yl]phenol (PHTPP), propylpyrazole triol (PPT), and diarylpropionitrile (DPN) were purchased from Tocris Bioscience (Ellisville, MO, USA). 1-[4-(6-bromo-benzo[1], [3]dioxol-5-yl)-3a,4,5,9b-tetrahydro-3H-cyclopenta[c]quinolin-8-yl]-ethanone (G-1) was bought from Cayman Chemical Co. (Ann Arbor, MI, USA).

### Cloning of Expression Vectors

Total RNA was extracted from MCF-7 cells (American Tissue Culture Collection, Manassas, VA, USA) by TRIzol reagent (Invitrogen, Carlsbad, CA, USA) and then reverse transcribed to cDNA by SuperScript first-strand synthesis system (Invitrogen) according to manufacturer’s instructions. Full-length cDNA transcripts of human ER66 (GenBank accession no. **NM_000125**), ER46 (**NM_000125**) and ER36 (**BX640939**) were synthesized by *Pfu* DNA polymerase (Stratagene, La Jolla, CA, USA) using forward and reverse oligonucleotide primers containing *EcoRI* and *XhoI* restriction sites, respectively (underlined). The forward primers are: 5′ GAATTC ATGACCATGACCCTCCACACCAAA 3′ for ER66, 5′ GAATTC GCCACCATGGCTA TGGAATCTGCCAAGAG 3′ for ER46 and 5′ GAATTC AAGGG AAGTATGGCTATGGAATCT 3′ for ER36; The reverse primers are: 5′ CTCGAG ACTGTGGCAGGGAAACCC 3′ for ER66 and ER46 and 5′ ATGCAAGTTCAGGATTCTCTTCTTTGCTTCTACATGTGAGATACCAGAA TTAAGCAAAAGAAT 3′ and 5′ CTCGAG ACACGAGGAAAC 3′ for ER36. The amplified products were cleaved with *EcoRI* (Invitrogen) and *XhoI* (Invitrogen), gel purified using the Wizard Plus minipreps DNA purification system (Promega, Madison, WI, USA), and subcloned into pEXP5-NT/TOPO vector and pcDNA3.1 (Invitrogen) for expression. The receptors were cloned in frame with an N-terminal polyhistidine region. All constructs were confirmed by sequencing (Invitrogen).

### Co-localization Study of mERs

Expression vectors of ER66, ER46 or ER36 were transfected to HEK293 cells with Optifect reagent (Invitrogen) according to manufacturer’s instructions. Transfected cells were incubated for at least 24 hours, and then plated on coverslips one to two days prior to the experiment. Cells were fixed with methanol for ten minutes and blocked in PBS with 3% BSA for two hours. Fixed cells were incubated with anti-VSVG antibody (1∶5000; Sigma) and anti-pan cadherin (1∶100; abcam) in PBS with 3% BSA at 4°C overnight. Cells were then incubated with secondary antibodies, Texas Red goat anti-mouse and Oregon Green 488 goat anti-rabbit (1∶160; Sigma), in PBS with 3% BSA for 1 hour at room temperature. Transfected cells were viewed under a LSM 510 META laser scanning confocal microscope (Carl Zeiss, Thornwood, NY, USA).

### Cell-free Expression of mERs

Human ER66, ER46 and ER36 proteins were synthesized using a eukaryotic TnT quick coupled transcription/translation system (Promega) composed of rabbit reticulocyte lysate with T7 RNA polymerase. The translation was initiated with incubation for 90 minutes at 30°C. Prokaryotic expressed receptor proteins were produced by the MembraneMax protein expression kit (Invitrogen) as described by the manufacturer. All reaction mixtures were supplemented with 2% (vol/vol) MembraneMax Reagent containing nanolipoprotein molecules (composed of mature human apoA1 and 1,2-dimyristoyl-*sn*-glycerol-3-phosphocholine (DMPC)) unless otherwise stated [37]. For inhibition of palmitoylation, 100 µM of 2-bromopalmitate was added to the reaction mixture.

### Western Blotting

Five microliter of expressed receptor proteins were incubated for 15 minutes in Laemmli buffer at 60°C and electrophoresed in a 10% SDS-PAGE gel. For native gel electrophoresis, native receptor proteins were mixed with native sample buffer and electrophoresed in a 7.5% Tris nondenaturing polyacrylamide gel (pH range 7.1 to 8.9). Proteins were transferred electrophoretically to polyvinylidene fluoride (PVDF) membranes (Bio-Rad Laboratories, Inc., Richmond, CA, USA) in a Tris-glycine transfer buffer with 20% methanol. The membranes were blocked by Tris buffered saline (TBS) containing 0.05% Tween and 5% fat-free powdered milk for 90 minutes and then incubated overnight in blocking buffer containing 1∶2500 anti-HisG-HRP antibody (Invitrogen). After washing, the signal was visualized by enhanced chemiluminescence (Amersham Biosciences, Arlington Heights, IL, USA) and exposed to x-ray film.

### Saturation Ligand Binding Assays

Receptor proteins (50 µg) were diluted in PBS and incubated with serial dilutions of [^3^H]-17β-estradiol in the presence or absence of a 300-fold excess of non-radioactive 17β-estradiol. All incubations were performed in glass test tubes at 4°C overnight. Bound and free radioligands were separated by filtration through a 25 mm glass microfiber GF/C filter (Whatman, Piscataway, NJ, USA) placed on a vacuum manifold (Hoefer, San Francisco, CA, USA). Filters were rapidly washed with ice-cold PBS and air-dried. Radioactivity of the filters was measured by the LS 6500 liquid scintillation counter (Beckman Coulter, Fullerton, CA, USA) after overnight incubation with ACS scintillation cocktail (Amersham, Piscataway, NJ, USA). Each assay point was run in duplicate, and the assays were repeated using two to three different batches of expressed receptor proteins. Specific binding was determined by subtracting non-specific binding (radioactivity of samples with an excess of unlabelled 17β-estradiol) from total binding. K_d_ were calculated as the free concentration of radioligands at half-maximal specific binding by fitting data to the Hill equation and by linear Scatchard transformation using Prism version 5.01 (GraphPad Software, La Jolla, CA, USA) [52], [53].

### Competitive Ligand Binding Assays

Receptor proteins were incubated with different concentrations (0.1 pM –10 µM) of the test compounds. [^3^H]-17β-estradiol was then added (to a final concentration of 100 pM). The reaction mixture was incubated at 4°C overnight in glass test tubes. Separation of bound and free radioligands and measurement of radioactivity were performed as described above. The data were fitted in a nonlinear four-parameter logistic model to estimate the half maximal inhibitory concentration value (IC_50_) [54]. Relative binding affinity (RBA) of each compound tested was calculated as the ratio of IC_50_ value for estrogen to that compound. The RBA value for estrogen was arbitrarily set at 100.
